# Generalized Bounded Linear Logic and its Categorical Semantics

**DOI:** 10.1007/978-3-030-71995-1_12

**Published:** 2021-03-23

**Authors:** Yōji Fukihara, Shin-ya Katsumata

**Affiliations:** 8 University of Oxford, Oxford, UK; 9 Sorbonne Université - LIP6, Paris, France; 10 Kyoto University, Kyoto, Japan; 11 National Institute of Informatics, Tokyo, Japan

**Keywords:** Linear Logic, Categorical Semantics, Linear Exponential Comonad, Graded Comonad

## Abstract

We introduce a generalization of Girard et al.’s BLL called GBLL (and its affine variant GBAL). It is designed to capture the core mechanism of dependency in BLL, while it is also able to separate complexity aspects of BLL. The main feature of GBLL is to adopt a multi-object pseudo-semiring as a grading system of the !-modality. We analyze the complexity of cut-elimination in GBLL, and give a translation from BLL with constraints to GBAL with positivity axiom. We then introduce *indexed linear exponential comonads* (*ILEC* for short) as a categorical structure for interpreting the ${!}$-modality of GBLL. We give an elementary example of ILEC using folding product, and a technique to modify ILECs with symmetric monoidal comonads. We then consider a semantics of BLL using the folding product on the category of assemblies of a BCI-algebra, and relate the semantics with the realizability category studied by Hofmann, Scott and Dal Lago.

## 1 Introduction

Girard's *linear logic* is a refinement of propositional logic by restricting weakening and contraction in proofs [15]. Linear logic also has an *of-course modality* !, which restores these structural rules to formulas of the form $!A$.

Later, Girard et al. extended the !-modality with quantitative information so that usage of !-modal formulas in proofs can be quantitatively controlled [16]. This extension, called *bounded linear logic* (BLL for short), is successfully applied to a logical characterization of P-time computations.

Their extension takes two steps. First, the !-modality is extended to the form $!_r A$, where the index $r$ is an element of a semiring [16, Section 2.4]. The index $r$ is called *grade* in modern terminology [11,13]. This extension and its variants have been employed in various logics and programming languages [7,30,14,26,28]. The categorical structure corresponding to $!_r A$ is identified as *graded linear exponential comonad* [7,13,22].

Second, the $!_r$-modality is further extended to the form $!_{x<p} A$, where $p$ is a polynomial (called *resource polynomial*) giving the upper bound of $x$ [16, Section 3]. The formula $!_{x<p} A$ also binds free occurrences of the resource variable

$x$ in resource polynomials in $A$. Therefore, in BLL, both formulas and resource polynomials depend on the values stored in free resource variables. This dependency mechanism significantly increases the expressiveness of BLL, leading to a characterization of P-time complexity.

This characterization result was later revisited through a *realizability semantics* of BLL [16,19,10]. Inside this semantics, however, mechanisms for controlling complexity of program execution are hard-coded, and it is not very clear which semantics structure realizes the dependency mechanism of BLL. This leads us to seek a logical and categorical understanding of BLL's dependency mechanism hidden underneath the complexity-related features, such as resource polynomials and computability constraints.

As a result of the quest, we propose a generalization of BLL called GBLL, and study its categorical semantics. The central idea of the generalization is to replace the grading semiring of the $!_r$-modality with a particular *multi-object pseudo-semiring* realized as a 2-category. Let us see how this replacement works. In GBLL, each formula is formed by deriving a judgment of the form $\Delta \vdash A$, where $\Delta$ is a set (called *index set*) and $A$ is a raw formula. We may think that such a well-formed formula $\Delta \vdash A$ denotes a $\Delta$-indexed family $\{[\![A]\!]_i\}_{i \in \Delta}$ of denotations. The formation rule for !-modal formula in GBLL is the following:

$$\frac{\Delta' \vdash A \quad f \in \mathbf{Set}(\Delta, (\Delta')^*)}{\Delta \vdash !_f A} \qquad ((\_)^*\text{: Kleene closure})$$

where the function $f$ abstractly represents dependency. This modality is enough to express the $!_{x<p}$-modality of BLL: we express the bindig $x < p$ under a resource variable context $\vec{y}$ as the *function* $f_p(\vec{y}) = (\vec{y}, 0) \cdots (\vec{y}, p(\vec{y}) - 1)$ that returns the list of environments extended with values less than $p(\vec{y})$. Then the denotation of the $!_f A$-modality is given by a variable-arity operator $D$. For each index $i \in \Delta$, the denotation is given by applying $D$ to the denotations obtained by mapping $A$ to list $f(i)$:

$$[\![!_f A]\!]_i = D([\![A]\!]_{j_1}, \cdots, [\![A]\!]_{j_n}) \text{ where } j_1 \cdots j_n = f(i).$$

A simple example of a variable-arity modal operator is the *folding product* $D(X_1, \cdots, X_n) = X_1 \otimes \cdots \otimes X_n$.

The pseudo-semiring structure on the class of functions of the form $\Delta \to (\Delta')^*$ is given as follows. For the multiplication $g \bullet f$, we adopt the *Kleisli composition* of the free monoid monad $(\_)^*$, while for the addition $f+g$, the pointwise concatenation $(f + g)(x) = f(x)g(x)$. However, these operations fail to satisfy one of the semiring axioms: $(f + g) \bullet h = f \bullet h + g \bullet h$. To fix this, we introduce (pointwise) list permutations as 2-cells between functions of type $\Delta \to (\Delta')^*$. These data form a 2-category **Idx**, which may be seen as a multi-object pseudo-semiring. Weakening, contraction, digging and dereliction in GBLL interact with these operations, much like the $!_r$-modality in [7].

We first study syntactic properties of GBLL. We introduce cut-elimination to GBLL and study its complexity property. It turns out that the proof technique used in BLL naturally extends to GBLL — as done in [16], we classify cuts into *reducible* and *irreducible* ones, introduce *proof weight*, and show that the reduction steps of reducible cuts will terminate in cubic time of proof weights. We also examine the expressive power of GBLL by giving a translation from an extension of BLL with *constraints* that are seen in Dal Lago et al.'s QBAL [10].

We next give a categorical semantics of GBLL. We introduce the concept of *indexed linear exponential comonad* (*ILEC*); it is an **Idx**-graded linear exponential comonad satisfying a commutativity condition with respect to an underlying indexed SMCCs. Then, we present a construction of ILEC from a symmetric monoidal closed category $\mathbb{C}$ with a symmetric monoidal comonad on it. We apply this construction to the case where $\mathbb{C}$ is the category of assemblies over a BCI algebra [2,20], and relate the semantics of GBLL with the constructed ILEC and the realizability category studied in [19,10].

*Acknowledgment* The first author was supported by JST ERATO HASUO Metamathematics for Systems Design Project (No. JPMJER1603). The authors are grateful to anonymous reviewers for comments, and Masahito Hasegawa, Naohiko Hoshino, Clovis Eberhart and Jérémy Dubut for fruitful discussions.

*Preliminaries* For a set $\Delta$, by $\Delta^*$ we mean the set of finite sequences of $\Delta$. The empty sequence is denoted by $()$. Juxtaposition of $\Delta^*$-elements denotes the concatenation of sequences. For $x \in \Delta^*$, by $|x|$ we mean the length of $x$. We identify a natural number $n$ and the set $\{0, \cdots, n-1\}$; note that $0 = \emptyset$. We also identify a sequence $x \in \Delta^*$ and the function "$\lambda i \in |x|$ . the $i$-th element of $x$".

## 2 Generalized Bounded Linear Logic

### 2.1 Indexing 2-Category

We first introduce a 2-category **Idx** (and its variant $\mathbf{Idx}_a$), which may be seen as a multi-object pseudo-semiring. It consists of the following data[3]: 0-cells are sets (called index sets), and the hom-category $\mathbf{Idx}(\Delta, \Delta')$, which is actually a groupoid, is defined by:

- An object (1-cell) is a function $f : \Delta \to (\Delta')^*$.
- A morphism (2-cell) from $f$ to $g$ in $\mathbf{Idx}(\Delta, \Delta')$ is a $\Delta$-indexed family of bijections $\{\sigma_x : |g(x)| \to |f(x)|\}_{x \in \Delta}$ such that $f(x)(\sigma_x(i)) = g(x)(i)$.

The identity 1-cell and the composition of 1-cells in **Idx** are denoted by $\mathrm{i}_\Delta$ and $(\bullet)$, respectively. The composition is defined by $(g \bullet f)(x) \stackrel{\text{def}}{=} g(y_1) \cdots g(y_n)$ where $y_1 \cdots y_n = f(x)$. The hom-category $\mathbf{Idx}(\Delta, \Delta')$ has a symmetric strict monoidal structure:

- the monoidal unit is the constant empty-sequence function $0(x) = ()$,

[3] This is a full sub-2-category of the Kleisli 2-category $\mathbf{CAT}_\mathcal{S}$, where $\mathcal{S}$ is the 2-monad of symmetric strict monoidal category [21].

– the tensor product of $f, g$, denoted by $f + g$, is defined by the index-wise concatenation $(f+g)(x) \stackrel{\text{def}}{=} f(x)g(x)$.

We write $J : \mathbf{Set} \to \mathbf{Idx}$ for the inclusion, namely $J\Delta = \Delta$ and $(Jf)(x) = f(x)$ (the singleton sequence).

**Proposition 2.1.** *The composition $\bullet$ is symmetric strong monoidal in each argument. Especially, we have*

$$f \bullet 0 = 0 \quad 0 \bullet f = 0 \quad f \bullet (g+h) = f \bullet g + f \bullet h \quad (f+g) \bullet h \cong f \bullet h + g \bullet h.$$

We also define $\mathbf{Idx}_a$ by replacing "bijection" in the definition of 2-cell of $\mathbf{Idx}$ with "injection". The hom-category $\mathbf{Idx}_a(\Delta, \Delta')$ has the 1-cell 0 as the terminal object, hence is a symmetric *affine* monoidal category.

## 2.2 Formulas and Proofs

**Definition of GBLL Formulas** We first fix a set-indexed sets $\{\mathcal{A}(\Delta)\}_{\Delta \in \mathbf{Set}}$ of atomic propositions. Formulas are defined by the following BNF:

$$A ::= a \star r \mid A \otimes A \mid A \multimap A \mid !_f A$$

where $a \in \mathcal{A}(\Delta)$ for some set $\Delta$, $r$ is a function (called *reindexing function*) and $f$ is a 1-cell in $\mathbf{Idx}$. Formula formation rules are introduced to derive the pair $\Delta \vdash A$ of an index set $\Delta$ and a formula $A$. They are defined as follows:

$$\frac{a \in \mathcal{A}(\Delta') \quad r \in \mathbf{Set}(\Delta, \Delta')}{\Delta \vdash a \star r} \qquad \frac{\Delta \vdash A \quad \Delta \vdash B}{\Delta \vdash A \otimes B} \qquad \frac{\Delta \vdash A \quad \Delta \vdash B}{\Delta \vdash A \multimap B}$$

$$\frac{\Delta' \vdash A \quad f \in \mathbf{Idx}(\Delta, \Delta')}{\Delta \vdash !_f A}$$

The formula $a \star r$ represents the atomic formula $a$ precomposed with a reindexing function $r$. We write $\mathbf{Fml}(\Delta) = \{A \mid \Delta \vdash A\}$.

We next introduce the reindexing operation on formulas.

**Definition 2.1.** *For a reindexing function $r \in \mathbf{Set}(\Delta, \Delta')$, we define the* reindexing operator $(\_)|_r : \mathbf{Fml}(\Delta') \to \mathbf{Fml}(\Delta)$ *along $r$ by*

$$a \star r|_{r'} \stackrel{\text{def}}{=} a \star (r \circ r'), \qquad (A \otimes B)|_r \stackrel{\text{def}}{=} A|_r \otimes B|_r,$$
$$(A \multimap B)|_r \stackrel{\text{def}}{=} A|_r \multimap B|_r, \qquad (!_f A)|_r \stackrel{\text{def}}{=} !_{f \bullet Jr} A.$$

*We routinely extend reindexing operators to sequences of formulas well-formed under a common index set.*

We quotient the set of well-formed formulas by the least congruent equivalence relation generated from the following binary relation:

$$\{(!_{Jr \bullet f} A, !_f(A|_r)) \mid r \in \mathbf{Set}(\Delta', \Delta''), f \in \mathbf{Idx}(\Delta, \Delta'), \Delta'' \vdash A\} \tag{2.1}$$

We see some formations of formulas in GBLL.

*Example 2.1.* Let us illustrate how a formula $!_{y<x^2}!_{z<x+y}A$ in BLL is represented in GBLL; here we assume that $x, y, z$ are the only resource variables used in this formula. We first introduce a notation. Let $E$ be a mathematical expression using variables $\mathbf{x}_1 \cdots \mathbf{x}_n$. Then by $[E]_n : \mathbb{N}^n \to (\mathbb{N}^{n+1})^*$ we mean the function

$$[E]_n(\vec{x}) = (\vec{x}, 0)(\vec{x}, 1) \cdots (\vec{x}, E[x_1/\mathbf{x}_1, \cdots, x_n/\mathbf{x}_n] - 1) \quad (\vec{x} \triangleq (x_1, \cdots, x_n) \in \mathbb{N}^n)$$

For instance, $[\mathbf{x}_1^2]_1(x) = (x, 0), \cdots, (x, x^2 - 1)$. Then from a well-formed formula $\mathbb{N}^3 \vdash A$, we obtain $\mathbb{N} \vdash !_{[\mathbf{x}_1^2]_1}!_{[\mathbf{x}_1+\mathbf{x}_2]_2}A$. Generalizing this, a BLL formula $!_{x<E}A$ containing resource variables $\mathbf{x}_1, \cdots, \mathbf{x}_n$ corresponds to the GBLL formula $!_{[E]_n}A$.

*Example 2.2.* We look at how we express the substitution of a resource polynomial $A[x := p(x_1, ..., x_n)]$. We define a function $\langle p\rangle_n : \mathbb{N}^n \to \mathbb{N}^{n+1}$ by

$$\langle p\rangle_n(x_1, ..., x_n) \stackrel{\text{def}}{=} (x_1, ..., x_n, p(x_1, ..., x_n)).$$

Then the reindexed formula $\mathbb{N}^n \vdash A|_{\langle p\rangle_n}$ corresponds to $A[x := p(x_1, \cdots, x_n)]$.

*Example 2.3.* We illustrate the equality between well-formed formulas. Consider a formula $\mathbb{N} \vdash A$ and a function $r \in \mathbf{Set}(\mathbb{N}^3, \mathbb{N})$. Then we equate formulas $\mathbb{N}^2 \vdash !_{[\mathbf{x}_1+\mathbf{x}_2]_2}(A|_r)$ and $\mathbb{N}^2 \vdash !_h A$, where $h \in \mathbf{Idx}(\mathbb{N}^2, \mathbb{N})$ is given by

$$h \stackrel{\text{def}}{=} Jr \bullet [\mathbf{x}_1 + \mathbf{x}_2]_2(x, y) = r(x, y, 0), \cdots, r(x, y, x + y - 1).$$

**Definition of GBLL Proofs** A *judgment* of GBLL is the form $\Delta \mid \Gamma \vdash A$, where $\Delta$ is an index set, $\Gamma$ is a sequence of formulas well-formed under $\Delta$, and $A$ is a well-formed formula under $\Delta$, respectively. The inference rules of GBLL are presented in Fig. 1. Similarly, we define GBAL to be the system obtained by replacing **Idx** in Fig. 1 with $\mathbf{Idx}_a$.

*Example 2.4.* We mimic a special case of the contraction rule in BLL

$$\frac{\Gamma, !_{x<x_i}A, !_{y<x_j}A\{^{x_i+y}/_x\} \vdash B}{\Gamma, !_{x<x_i+x_j}A \vdash B}$$

See also (!C)-rule of CBLL in Section 3.2. We use the *shift function* $s_{n,i} \in \mathbf{Set}(\mathbb{N}^{n+1}, \mathbb{N}^{n+1})$ defined by $s_{n,i}(x_1, \cdots, x_n, y) \stackrel{\text{def}}{=} (x_1, \cdots, x_n, x_i + y)$. Then we easily see $[\mathbf{x}_i]_n + Js_{n,i} \bullet [\mathbf{x}_j]_n = [\mathbf{x}_i + \mathbf{x}_j]_n$. By contraction rule of GBLL, we obtain the following derivation for well-formed formulas $\mathbb{N}^{n+1} \vdash A$ and $\mathbb{N}^n \vdash B$, mimicking the contraction of BLL:

$$\frac{!_{[\mathbf{x}_i]_n}A, !_{[\mathbf{x}_j]_n}(A|s_{n,i}) \vdash B}{!_{[\mathbf{x}_i+\mathbf{x}_j]_n}A = !_{[\mathbf{x}_i]_n+Js_{n,i}\bullet[\mathbf{x}_j]_n}A \vdash B}$$

Here, we use the formula equality $!_{Js_{n,i}\bullet[\mathbf{x}_j]_n}A = !_{[\mathbf{x}_j]_n}(A|s_{n,j})$.

$$\frac{\Delta \vdash A}{\Delta \mid A \vdash A}\text{ (Ax) Axiom} \qquad \frac{\Delta \mid \Gamma, X, Y, \Gamma' \vdash A}{\Delta \mid \Gamma, Y, X, \Gamma' \vdash A}\text{ (Exch) Exchange}$$

$$\frac{\Delta \mid \Gamma_1 \vdash A \quad \Delta \mid \Gamma_2, A \vdash B}{\Delta \mid \Gamma_1, \Gamma_2 \vdash B}\text{ (Cut)}$$

$$\frac{\Delta \mid \Gamma, X, Y \vdash A}{\Delta \mid \Gamma, X \otimes Y \vdash A}\text{ }(\otimes\text{L}) \qquad \frac{\Delta \mid \Gamma_1 \vdash X \quad \Delta \mid \Gamma_2 \vdash Y}{\Delta \mid \Gamma_1, \Gamma_2 \vdash X \otimes Y}\text{ }(\otimes\text{R})$$

$$\frac{\Delta \mid \Gamma_1 \vdash X \quad \Delta \mid \Gamma_2, Y \vdash B}{\Delta \mid \Gamma_1, \Gamma_2, X \multimap Y \vdash B}\text{ }(\multimap\text{L}) \qquad \frac{\Delta \mid \Gamma, X \vdash Y}{\Delta \mid \Gamma \vdash X \multimap Y}\text{ }(\multimap\text{R})$$

$$\frac{\Delta \mid \Gamma \vdash B}{\Delta \mid \Gamma, !_0 A \vdash B}\text{ (!W) Weakening} \qquad \frac{\Delta \mid \Gamma, A \vdash B}{\Delta \mid \Gamma, !_{\mathrm{id}} A \vdash B}\text{ (!D) Dereliction}$$

$$\frac{\Delta \mid \Gamma, !_g A \vdash B \quad \sigma \in \mathbf{Idx}(\Delta, \Delta')(f, g)}{\Delta \mid \Gamma, !_f A \vdash B}\text{ (!F) !-Functor}$$

$$\frac{\Delta \mid \Gamma, !_{f_1} A, !_{f_2} A \vdash B}{\Delta \mid \Gamma, !_{f_1 + f_2} A \vdash B}\text{ (!C)Contraction}$$

$$\frac{\Delta' \mid !_{g_1} A_1, \cdots, !_{g_k} A_k \vdash B \quad f \in \mathbf{Idx}(\Delta, \Delta')}{\Delta \mid !_{g_1 \bullet f} A_1, \cdots, !_{g_k \bullet f} A_k \vdash !_f B}\text{ (P!) Composition}$$

**Fig. 1.** GBLL Proof Rules

*Example 2.5.* The reindexing operator can be extended to proofs. Let $r$ be a reindexing function in $\mathbf{Set}(\Delta, \Delta')$. Reindexing of the axiom rule $\Delta' \mid A \vdash A$, by $r$ is the axiom rule $\Delta \mid A|_r \vdash A|_r$. Reindexing of other rules except (P!) can be easily defined—the judgment $\Delta' \mid \Gamma \vdash A$ in each rule is replaced with $\Delta \mid \Gamma|_r \vdash A|_r$ by reindexing. For (P!) rule, reindexing by $r$ is given as follows:

$$\frac{\Delta'' \mid !_{g_1} A_1, \cdots, !_{g_k} A_k \vdash B \qquad f \bullet Jr \in \mathbf{Idx}(\Delta, \Delta'')}{\Delta \mid (!_{g_1 \bullet f} A_1)|_r, \cdots, (!_{g_k \bullet f} A_k)|_r \vdash (!_f B)|_r}$$

*Remark 2.1.* In this paper, indexing 2-category is either $\mathbf{Idx}$ or $\mathbf{Idx}_a$. Allowing more general indexing 2-categories in GBLL is a future work. In his PhD thesis, Breuvart designed a linear logic similar to GBLL upon an abstract indexing mechanism called *dependent semirings* [5, Definition 3.2.4.5]. It consists of categories $(\mathcal{S}, \mathcal{U})$ such that 1) each hom-set in $\mathcal{S}$ carries a (not necessarily commutative) ordered monoid structure $(0, +)$ and the composition of $\mathcal{S}$ distributes over $0, +$, and 2) $\mathcal{U}$ acts on $\mathcal{S}$ from both sides. Roughly speaking, $\mathcal{S}$ and $\mathcal{U}$ corresponds to our $\mathbf{Idx}^{\mathrm{op}}$ and $\mathbf{Set}^{\mathrm{op}}$, respectively. We expect that a unification of dependent semirings and 2-categories $\mathbf{Idx}, \mathbf{Idx}_a$ would yield a suitable generalization of indexing categories for GBLL. This generalization will subsume the non-graded linear logic, and allow us to compare GBLLs over different idexing categories.

### 2.3 Complexity of Cut-Elimination in GBLL

By a similar discussion to BLL [16], instances of Cut inference are divided in two classes: *reducible* cuts and *irreducible* cuts. We define the *weight* of proof $|\pi|$ for each proof $\pi \triangleright \Delta \mid \Gamma \vdash A$ and *reduction steps* of proofs, such that every reduction steps will terminate, for each index $\delta \in \Delta$, in polynomial steps of $|\pi|(\delta)$.

**Definition 2.2.** *[16, Appedix A] In GBLL (resp. GBAL) proofs, an instance of the Cut inference is* irreducible *if there are at least one Composition rule below it or if its left premise is obtained by a Composition rule with nonempty context and the other premise is obtained by a Weakening, !-Functor, Dereliction, Contraction or Composition inference. A* reducible *cut is Cut inferences that is not irreducible.*

The definition of (ir)reducibility and weight is diverted from Girard's paper. Therefore, our system inherits from BLL the conditions under which cuts can be reduced. See also Section 2.4 in [16].

**Definition 2.3.** *A GBLL or GBAL proof is* irreducible *if it contains only irreducible cut inferences.*

Following [16], we introduce the concept of *weight* of a proof. It is a function $|\pi| : \Delta \to \mathbb{N}$ assigning a weight number $|\pi|(\delta)$ to a proof $\pi$ at an index $\delta \in \Delta$. The weight number never increases at any reduction step of Cut in $\pi$. In the original BLL, weights are expressed by resource polynomials, while here, they are generalized to arbitrary functions. We remark that weights of the proofs involving Composition rules, which introduce $!_f$ modality, use the length of the lists constructed by $f$.

**Definition 2.4.** *For a given proof $\pi \triangleright \Delta \mid \Gamma \vdash A$ of GBLL or GBAL, the* weight *of $\pi$ is a function $|\pi| : \Delta \to \mathbb{N}$ inductively defined as follows. A) When $\Delta = \emptyset$, $|\pi|$ is the evident function. B) When $\Delta \neq \emptyset$, $|\pi|$ is defined by the following rules:*

1. *For an Axiom rule $\pi \triangleright \Delta \mid A \vdash A$, $|\pi|(\delta) \stackrel{\text{def}}{=} 1$.*
2. *If $\pi$ is obtained from $\pi'$ by a unary rule except Contraction and Composition, $|\pi|(\delta) \stackrel{\text{def}}{=} |\pi'|(\delta) + 1$.*
3. *If $\pi$ is obtained from $\pi_1$ and $\pi_2$ by a binary rule except Cut, $|\pi|(\delta) \stackrel{\text{def}}{=} |\pi_1|(\delta) + |\pi_2|(\delta) + 1$.*
4. *If $\pi$ is obtained from $\pi_1$ and $\pi_2$ by a Cut rule, $|\pi|(\delta) \stackrel{\text{def}}{=} |\pi_1|(\delta) + |\pi_2|(\delta)$.*
5. *If $\pi$ is obtained from $\pi'$ by a Contraction rule, $|\pi|(\delta) \stackrel{\text{def}}{=} |\pi'|(\delta) + 2$*
6. *If $\pi$ is obtained from $\pi'$ by a Composition rule, such as*

$$\pi \triangleright \frac{\begin{array}{c} \vdots \pi' \\ \Delta' \mid !_{\alpha_1} A_1, \cdots, !_{\alpha_k} A_k \vdash B \end{array}}{\Delta \mid !_{\alpha_1 \bullet f} A_1, \cdots, !_{\alpha_k \bullet f} A_k \vdash !_f B}$$

*then* $|\pi|(\delta) \stackrel{\text{def}}{=} \sum_{\gamma \in f(\delta)} (|\pi'|(\gamma) + 2k + 1) + k + 1$*. Note that the summation* $\sum_{\gamma \in f(\delta)}$ *scans all elements in the list* $f(\delta)$*, hence the weight depends on the length of* $f(\delta)$*.*

**Theorem 2.1.** *For every proof* $\pi \rhd \Delta \mid \Gamma \vdash A$ *and every* $\delta \in \Delta$*, reduction steps of reducible cuts will terminate in at most* $(|\pi|(\delta))^3$ *steps.*

*Proof (sketch).* The proof is almost the same as Section 2.2 and Appendix A of [16], except for the definition of the weight. Suppose that $\pi$ one-step reduces into $\pi'$. From the definition of the weight, either 1) for all index $\delta \in \Delta$, the weight decreases (that is, $|\pi|(\delta) > |\pi'|(\delta)$), or 2) for all index $\delta \in \Delta$, the weight keeps (that is, $|\pi|(\delta) = |\pi'|(\delta)$). The reduction of the former type is called symmetric or axiom reduction [16, Section 2.2.1 and 2.2.2], while the latter commutative reduction [16, Section 2.2.3].

In the case where the weight keeps, we introduce another measure called the *cut size* $\|\pi\| : \Delta \to \mathbb{N}$ of a proof $\pi$. Its definition is the same as the definition of weight except for Cut rule. For a proof $\pi$ obtained by Cut rule from $\pi_1$ and $\pi_2$, the cut size $\|\pi\|(\delta)$ is defined to be $\|\pi_1\|(\delta) + \|\pi_2\|(\delta) + |\pi_1|(\delta) + |\pi_2|(\delta)$.

In each commutative reduction from $\pi$ to $\pi'$ the cut size decrease at all index (that is, for all $\delta \in \Delta$, $||\pi||(\delta) > ||\pi'||(\delta)$), and the cut size is at most the square of the weight (that is, for all $\delta \in \Delta, ||\pi||(\delta) \leq (|\pi|(\delta))^2$). Therefore, the total number of steps is at most the cube of the weight. □

The number of reduction steps of a proof $\pi$ and its weight depend on the length of lists computed by the **Idx**-morphisms occurring in $\pi$. However, to discuss the actual time complexity of cut-elimination, we further need to take into account the time complexity of the computation of **Idx**-morphisms. This would be achieved by looking at a subcategory of **Idx** computable within a certain time complexity. We leave this argument of analyzing the actual time complexity of cut-elimination as a future work.

## 3 Translation from Constrained BLL

We show that GBLL can express BLL via a translation. This translation is actually given to variants of these calculi, namely from *BLL with constraints* (called CBLL) to *GBAL with positivity axioms* (called $\mathsf{GBAL}^+$).

CBLL is an extension of BLL with *constraints*, which are one of the features of Dal Lago and Hofmann's QBAL [10]. Constraints explicitly specify conditions imposed on resource variables, and it is natural to explicitly maintain these conditions throughout proofs. We also remark that in CBLL, weakening of !-formulas $!_{x<p+q}A \multimap !_{x<p}A$ is allowed, and atomic formulas are assumed to satisfy the positivity property (3.1).

$\mathsf{GBAL}^+$ is designed for a sound translation from CBLL. Recall that GBAL is an extension of GBLL with weakening $!_{f+g}A \multimap !_f A$ on !-formulas. Then $\mathsf{GBAL}^+$ is a further extension of GBAL with the following positivity axioms of atomic formulas: for every $n$-ary atomic formula $a \in \mathcal{A}$ in CBLL, we introduce an atomic formula $[a] \in \mathcal{A}(\mathbb{N}^n)$ to GBAL together with the axiom:

$$V_{\mathscr{C}}(F) \mid \emptyset \vdash [a] \star \langle p_1, \cdots, p_n \rangle \multimap [a] \star \langle q_1, \cdots, q_n \rangle \quad (\forall i. p_i \sqsubseteq_{\mathscr{C}} q_i).$$

Here the definition of each notation is given in Section 3.1 and 3.3. Positivity axiom induces proofs $V_{\mathscr{C}}(F) \mid A' \vdash A$ for every two formulas $A, A'$ such that $A' \sqsubseteq_{\mathscr{C}} A$ (the relation $\sqsubseteq_{\mathscr{C}}$ for formulas is defined in Section 3.2).

### 3.1 Resource Polynomials and Constraints

We introduce basic concepts around CBLL, referring to its super-logic QBAL [10]. We put a reference in the beginning of each paragraph when the contents come from QBAL in [10].

[10, Definition 2.1] Given a countably infinite set $\mathcal{RV}$ of *resource variables*, a *resource monomial* over $\mathcal{RV}$ is a finite product of binomial coefficients $\prod_{i=1}^{m} \binom{x_i}{n_i}$, where the resource variables $x_1, \cdots, x_m$ are distinct and $n_1, \cdots, n_m \in \mathbb{N}$ are natural numbers. A *resource polynomial* over $\mathcal{RV}$ is a finite sum of resource monomials. We write 1 as $\binom{x}{0}$ and $x$ as $\binom{x}{1}$ for short. Each positive natural number $n$ denotes a resource polynomial $1 + 1 + \cdots + 1$. Resource polynomials are closed under sum, product, bounded sum and composition [10, Lemma 2.2].

[10, Definition 2.3] A *constraint* is an inequality $p \leq q$, where $p$ and $q$ are resource polynomials. We abbreviate $p+1 \leq q$ as $p < q$. A constraint $p \leq q$ *holds* (written $\vDash p \leq q$) if it is true in the standard model. A *constraint set* (denoted with $\mathscr{C}$, $\mathscr{D}$) is a finite set of constraints. A constraint $p \leq q$ *is a consequence* of a constraint set $\mathscr{C}$ (written $\mathscr{C} \vDash p \leq q$) if $p \leq q$ is a logical consequence of $\mathscr{C}$. For every constraint sets $\mathscr{C}$ and $\mathscr{D}$, we write $\mathscr{C} \vDash \mathscr{D}$ iff $\mathscr{C} \vDash p \leq q$ for every constraint $p \leq q$ in $\mathscr{D}$. For each constraint set $\mathscr{C}$, we define an order $\sqsubseteq_{\mathscr{C}}$ on resource polynomials by $p \sqsubseteq_{\mathscr{C}} q$ iff $\mathscr{C} \vDash p \leq q$.

[10, Definition 2.3] We define the polarity of occurrences of free resource variables. For a constraint $p \leq q$, we say that an occurrence of a resource variable $x$ in $p$ is called *negative*, while the one in $q$ is called *positive*.

### 3.2 Formulas and Inference Rules of CBLL

Let $\mathcal{A}$ be a set of atomic formulas and assume that each atomic formula $a \in \mathcal{A}$ is associated with an arity $\mathrm{ar}(a)$. Formulas of CBLL are defined by:

$$A, B ::= \quad a(p_1, \cdots, p_{\mathrm{ar}(a)}) \mid A \otimes B \mid A \multimap B \mid !_{x<p} A$$

where $p$ in the formula $!_{x<p}A$ satisifes $x \notin \mathrm{FV}(p)$.

[10, Definition 2.6] Each occurrence of a free resource variable in a formula is classified into *positive* or *negative*. Below we inductively define a *positive occurrence* of a resource variable. An occurrence of $x$ in:

- $a(p_1, \cdots, p_{\mathrm{ar}(a)})$ is always positive.
- $A \otimes B$ is positive iff it is in $A$ and positive, or so in $B$.

$$\frac{A \sqsubseteq_{\mathscr{C}} B}{A \vdash_{\mathscr{C}} B}\ (\mathrm{Ax}) \qquad \frac{\Gamma \vdash_{\mathscr{C}} A \quad \mathscr{D} \vDash \mathscr{C}}{\Gamma \vdash_{\mathscr{D}} A}\ (\mathrm{Str}) \qquad \frac{\Gamma \vdash_{\mathscr{C}} B}{\Gamma, !_{x<0} A \vdash_{\mathscr{C}} B}\ (!\mathrm{W})$$

$$\frac{A\{0/x\}, \Gamma \vdash_{\mathscr{C}} B}{!_{x<1} A, \Gamma \vdash_{\mathscr{C}} B}\ (!\mathrm{D}) \qquad \frac{\Gamma, !_{x<p} A, !_{y<q} A\{p+y/x\} \vdash_{\mathscr{C}} B}{\Gamma, !_{x<p+q} A \vdash_{\mathscr{C}} B}\ (!\mathrm{C})$$

$$\frac{A_1, \cdots, A_n \vdash_{\mathscr{C} \cup \{x<p\}} B \quad x \notin \mathrm{FV}(\mathscr{C})}{!_{x<p} A_1, \cdots, !_{x<p} A_n \vdash_{\mathscr{C}} !_{x<p} B}\ (!\mathrm{P})$$

$$\frac{!_{y<p} !_{z<q\{y/w\}} A \left\{ \left(z + \sum_{w<y} q(w)\right) / x \right\}, \Gamma \vdash_{\mathscr{C}} B}{!_{x< \sum_{w<p} q(w)} A, \Gamma \vdash_{\mathscr{C}} B}\ (!\mathrm{N})$$

**Fig. 2.** Inference Rules for CBLL ($\otimes$ and $\multimap$ are omitted)

- $A \multimap B$ is positive iff it is in $A$ and negative, or it is in $B$ and positive.
- $!_{x'<p} A$ is positive iff it is in $A$ and positive. We remark that an occurrence of a free resource variable in $p$ is counted as negative in $!_{x'<p} A$.

[10, Definition 2.8] We extend the order $\sqsubseteq_{\mathscr{C}}$ on resource polynomials to the one on CBLL formulas.

$$\begin{aligned}
&a(p_1, \cdots, p_{\mathrm{ar}(a)}) \sqsubseteq_{\mathscr{C}} a(q_1, \cdots, q_{\mathrm{ar}(a)}) \text{ iff } \forall i. p_i \sqsubseteq_{\mathscr{C}} q_i \\
&A \otimes B \sqsubseteq_{\mathscr{C}} C \otimes D \text{ iff } (A \sqsubseteq_{\mathscr{C}} C) \wedge (B \sqsubseteq_{\mathscr{C}} D) \\
&A \multimap B \sqsubseteq_{\mathscr{C}} C \multimap D \text{ iff } (C \sqsubseteq_{\mathscr{C}} A) \wedge (B \sqsubseteq_{\mathscr{C}} D) \\
&!_{x<p} A \sqsubseteq_{\mathscr{C}} !_{x<q} B \text{ iff } (q \sqsubseteq_{\mathscr{C}} p) \wedge (x \notin \mathrm{FV}(\mathscr{C})) \wedge (A \sqsubseteq_{\mathscr{C} \cup \{x<q\}} B)
\end{aligned} \tag{3.1}$$

[10, Section 2.3] A CBLL *judgment* is an expression $\Gamma \vdash_{\mathscr{C}} A$, where $\mathscr{C}$ is a constraint set, $\Gamma$ is a multiset of formulas and $A$ is a formula. A judgment $\Gamma \vdash_{\mathscr{C}} A$ means that $A$ is a consequence of $\Gamma$ under the constraints $\mathscr{C}$.

*Inference rules* (Fig. 2) are almost the same as those of QBAL; we omit the rules for $\otimes, \multimap$ and Cut. Note that weakening is restricted to !-formulas. Every BLL proof of $\Gamma \vdash A$ can be translated to a CBLL proof of $\Gamma \vdash_{\emptyset} A$.

### 3.3 Translation into GBAL$^+$

As mentioned at the beginning of Section 3, we will give a translation from CBLL to GBAL$^+$. When translating a CBLL proof $\Gamma \vdash_{\mathscr{C}} A$, we also need to supply a set $F$ of free resource variables satisfying $F \supseteq \mathrm{FV}(\Gamma) \cup \mathrm{FV}(A) \cup \mathrm{FV}(\mathscr{C})$. Then the translation of the proof of $\Gamma \vdash_{\mathscr{C}} A$ yields a proof of $V_{\mathscr{C}}(F) \mid [\Gamma]^{(F;\mathscr{C})} \vdash [A]^{(F;\mathscr{C})}$ in GBAL$^+$.

**For Constraints** We define an *environment* over a finite set $F$ of resource variables to be a function from $F$ to $\mathbb{N}$; by $V(F)$ we mean the set of environments over $F$. Given an environment $\rho \in V(F)$ and a resource variable $x \notin F$ and $n \in \mathbb{N}$, by $\rho\{x \mapsto n\}$ we mean the environment over $F \cup \{x\}$ that extends $\rho$

with a mapping $x \mapsto n$. Given a resource polynomial $p$ such that $FV(p) \subseteq F$, by $[\![p]\!] : V(F) \to \mathbb{N}$ we mean the function that evaluates the resource polynomial $p$ under a given environment. For resource polynomials $p_1, \cdots, p_n$ such that $\mathrm{FV}(p_i) \subseteq F$, we give a function $\langle p_1, \cdots, p_n \rangle : (V(F)) \to \mathbb{N}^n$ by $\langle p_1, \cdots, p_n \rangle \rho = ([\![p_1]\!]\rho, \cdots, [\![p_n]\!]\rho)$.

Let $\rho \vDash p \leq q$ denote $[\![p]\!]\rho \leq [\![q]\!]\rho$ for a constraint $p \leq q$ with a set $F$ of free resource variables (such that $\mathrm{FV}(p) \cup \mathrm{FV}(q) \subseteq F$) and for an environment $\rho \in V(F)$. For a subset $S \subset V(F)$ and for a constraint set $\mathscr{C}$, $S \vDash \mathscr{C}$ is also defined similarly: for every $\rho \in S$ and for every $p \leq q \in \mathscr{C}$, $\rho \vDash p \leq q$. Given a constraint set $\mathscr{C}$ and a set $F$ of resource variables such that $\mathrm{FV}(\mathscr{C}) \subseteq F$, let a set $V_{\mathscr{C}}(F)$ and a function $\iota_{F,\mathscr{C}} : V_{\mathscr{C}}(F) \to V(F)$ be given by:

$$V_{\mathscr{C}}(F) \stackrel{\text{def}}{=} \{\rho \in V(F) \mid \rho \vDash \mathscr{C}\}, \qquad \iota_{F,\mathscr{C}}(\rho) \stackrel{\text{def}}{=} \rho.$$

For a resource polynomial $p$, a free resource variable $x$ such that $x \notin \mathrm{FV}(p)$, a constraint set $\mathscr{C}$ and a set $F$ of resource variables such that $\mathrm{FV}(p) \cup \mathrm{FV}(\mathscr{C}) \subseteq F$, we introduce a map $[x < p]_{(F,\mathscr{C})} : V_{\mathscr{C}}(F) \to V_{\mathscr{C} \cup \{x<p\}}(F \cup \{x\})^*$ by

$$[x < p]_{(F,\mathscr{C})}\rho \stackrel{\text{def}}{=} \rho\{x \mapsto 0\}, \rho\{x \mapsto 1\}, \cdots, \rho\{x \mapsto ([\![p]\!]\rho - 1)\}$$

**For Formulas** Given a $\mathsf{CBLL}$ formula $A$, a constraint set $\mathscr{C}$ and a set of resource variables $F$ such that $F \supseteq \mathrm{FV}(A) \cup \mathrm{FV}(\mathscr{C})$, the translation $[A]^{(F;\mathscr{C})}$ of a well-formed formula $V_{\mathscr{C}}(F) \vdash A$ is defined inductively as follows:

$$[a(p_1, ..., p_n)]^{(F;\mathscr{C})} \stackrel{\text{def}}{=} [a] \star (\langle p_1, ..., p_n \rangle \circ \iota_{F,\mathscr{C}})$$
$$[A \otimes B]^{(F;\mathscr{C})} \stackrel{\text{def}}{=} [A]^{(F;\mathscr{C})} \otimes [B]^{(F;\mathscr{C})}$$
$$[A \multimap B]^{(F;\mathscr{C})} \stackrel{\text{def}}{=} [A]^{(F;\mathscr{C})} \multimap [B]^{(F;\mathscr{C})}$$
$$[!_{x<p}A]^{(F;\mathscr{C})} \stackrel{\text{def}}{=} !_{[x<p]_{(F,\mathscr{C})}}[A]^{(F \cup \{x\};\mathscr{C} \cup \{x<p\})}$$

**For Proofs** To give a translation of proofs, we define another notation. For a resource polynomial $p, q$, a set $F$ of resource variables and a constraint set $\mathscr{C}$ such that $\mathrm{FV}(p) \cup \mathrm{FV}(\mathscr{C}) \subseteq F$, a set $[p, q)^{(F,\mathscr{C})}$ of environments is defined by

$$[p, q)^{(F,\mathscr{C})} = \{\rho \in V(F \cup \{t\}) \mid \rho \vDash \mathscr{C}, [\![p]\!](\rho) \leq \rho(t) < [\![p + q]\!]\rho\}$$

here $t$ is a "fresh" resource variable such that $t \notin F$.

Given a proof $\pi \triangleright \Gamma \vdash_{\mathscr{C}} A$, a translation $[\pi]^{(F;\mathscr{C})} \triangleright V_{\mathscr{C}}(F) \mid [\Gamma]^{(F;\mathscr{C})} \vdash [A]^{(F;\mathscr{C})}$ is defined inductively on the structure of the proof:

- For Axiom rule, we can prove $V_{\mathscr{C}}(F) \mid [A]^{(F;\mathscr{C})} \vdash [B]^{(F;\mathscr{C})}$ for formulas $A, B$ such that $A \sqsubseteq_{\mathscr{C}} B$.
- For rules (Cut), ($\otimes$L), ($\otimes$R), ($\multimap$L), ($\multimap$R) and (!W), the translation is simple replacement of each formula $A$ with $[A]^{(F;\mathscr{C})}$.

– For (Str) rule, we have a map $r \in \mathbf{Set}(V_{\mathscr{D}}(F), V_{\mathscr{C}}(F))$. Then the translation is given as reindexed proof $[\pi']^{(F;\mathscr{C})}|_r$ of the translation $[\pi']^{(F;\mathscr{C})}$ of the premise.
– For (!D) rule, the premise is translated to $V_{\mathscr{C}}(F) \mid A', [\Gamma]^{(F;\mathscr{C})} \vdash [B]^{(F;\mathscr{C})}$, where $A' = [A]^{(F\cup\{x\};\mathscr{C}\cup\{x<1\})}|_r$ and $r$ is a map such that $Jr = [x < 1]_{(F,\mathscr{C})}$.
– For (!C) rule, we define a morphism $s^{(F;\mathscr{C})}_{p,q}$ in $\mathbf{Idx}_a$ and functions $r^{(F;\mathscr{C})}_{p,q}$, $i_1^{(p,q;F;\mathscr{C})}$, $i_2^{(p,q;F;\mathscr{C})}$ ($s, r, i_1$ and $i_2$ for short) by

$$
\begin{aligned}
s^{(F;\mathscr{C})}_{p,q}: \quad & V_{\mathscr{C}}(F) \to [p,q)^{(F,\mathscr{C})} \\
& \rho \mapsto \rho\{t \mapsto [\![p]\!]\rho\}, \cdots, \rho\{t \mapsto ([\![p+q]\!]\rho - 1)\} \\
r^{(F;\mathscr{C})}_{p,q}: \quad & [p,q)^{(F,\mathscr{C})} \xrightarrow{\sim} V_{\mathscr{C}\cup\{y<q\}}(F \cup \{y\}) \\
& \rho\{t \mapsto ([\![p]\!]\rho + k)\} \mapsto \rho\{y \mapsto k\} \\
i_1^{(p,q;F;\mathscr{C})}: \quad & V_{\mathscr{C}\cup\{x<p\}}(F \cup \{x\}) \to V_{\mathscr{C}\cup\{x<p+q\}}(F \cup \{x\}) \\
& \rho\{x \mapsto k\} \mapsto \rho\{x \mapsto k\} \\
i_2^{(p,q;F;\mathscr{C})}: \quad & [p,q)^{(F,\mathscr{C})} \to V_{\mathscr{C}\cup\{x<p+q\}}(F \cup \{x\}) \\
& \rho\{t \mapsto ([\![p]\!]\rho + k)\} \mapsto \rho\{x \mapsto [\![p]\!]\rho + k\}
\end{aligned}
$$

They satisfy $!_{[x<p]}[A]^{(F\cup\{x\};\mathscr{C}\cup\{x<p\})} = !_{[x<p]}([A]^{(F\cup\{x\};\mathscr{C}\cup\{x<p+q\})}|_{i_1})$ and $!_{[y<q]}[A\{{}^{p+y}/_{x}\}]^{(F\cup\{y\};\mathscr{C}\cup\{y<q\})} = !_{Jr\bullet s}([A]^{(F\cup\{x\};\mathscr{C}\cup\{x<p+q\})}|_{i_2\circ r^{-1}})$.
Then the conclusion of (!C) is obtained:

$$V_{\mathscr{C}}(F) \mid [\Gamma]^{(F;\mathscr{C})}, !_{(Ji_i\bullet[x<p])+(Ji_2\bullet s)}[A]^{(F\cup\{x\};\mathscr{C}\cup\{x<p+q\})} \vdash [B]^{(F;\mathscr{C})}.$$

– For (!P) rule, let $F' = F \cup \{x\}$ and $\mathscr{C}' = \mathscr{C} \cup \{x < p\}$. We can prove the translated conclusion from the translated premise by the following proof:

$$
\dfrac{\dfrac{\dfrac{V_{\mathscr{C}'}(F') \mid [A_1]^{(F';\mathscr{C}')}, \cdots, [A_n]^{(F';\mathscr{C}')} \vdash [B]^{(F';\mathscr{C}')}}{n \text{ times (!D)'s} \ \vdots}}{V_{\mathscr{C}'}(F') \mid !_{\mathrm{id}}[A_1]^{(F';\mathscr{C}')}, \cdots, !_{\mathrm{id}}[A_n]^{(F';\mathscr{C}')} \vdash [B]^{(F';\mathscr{C}')}}}{V_{\mathscr{C}}(F) \mid !_{[x<p]}[A_1]^{(F';\mathscr{C}')} \cdots !_{[x<p]}[A_n]^{(F';\mathscr{C}')} \vdash !_{[x<p]}[B]^{(F';\mathscr{C}')}}
$$

– For (!N) rule, we define index sets $\Delta_0, \Delta_1, \Delta_2$ and constraints $\mathscr{C}_0, \mathscr{C}_1, \mathscr{C}_2$ by

$$
\begin{aligned}
\mathscr{C}_0 &= \mathscr{C} \cup \{y < p\} & \Delta_0 &= V_{\mathscr{C}_0}(F \cup \{y\}) \\
\mathscr{C}_1 &= \mathscr{C} \cup \{y < p, z < q\{{}^{y}/_{w}\}\} & \Delta_1 &= V_{\mathscr{C}_1}(F \cup \{y, z\}) \\
\mathscr{C}_2 &= \mathscr{C} \cup \{x < \sum_{w<p} q(w)\} & \Delta_2 &= V_{\mathscr{C}_2}(F \cup \{x\})
\end{aligned}
$$

There is an isomorphism $r \in \mathbf{Set}(\Delta_1, \Delta_2)$, and it holds an equation $[z < q\{{}^{y}/_{w}\}]_{(F\cup\{y\},\mathscr{C}_0)} \bullet [y < p]_{(F,\mathscr{C})} = Jr^{-1} \bullet [x < \sum_{w<p} q(w)]_{(F,\mathscr{C})}$. Therefore, (!N) rule can be translated to the following provable judgment:

$$V_{\mathscr{C}}(F) | !_{[x<\sum_{w<p} q]}[A]^{(F\cup\{x\};\mathscr{C}_2)} \vdash !_{[y<p]}!_{[z<q\{y/w\}]}[A\{{}^{z+\sum_{w<y} q}/_{x}\}]^{(F\cup\{y,z\};\mathscr{C}_1)}$$

Since every $\mathsf{BLL}$ proof $\Gamma \vdash A$ can be translated to a $\mathsf{CBLL}$ proof $\Gamma \vdash_{\emptyset} A$, it can further be translated to a $\mathsf{GBAL}^+$ proof $V_{\emptyset}(F) \mid [\Gamma]^{(F;\emptyset)} \vdash [A]^{(F;\emptyset)}$.

## 4 Categorical Semantics for GBLL

We give a categorical semantics of GBLL. First, notice that each index set $\Delta$ determines a multiplicative linear logic under $\Delta$. We model this situation by a *set-indexed symmetric monoidal closed categories*, given by a functor $C : \mathbf{Set}^{op} \to \mathbf{SMCC}_{\text{strict}}$. That is, for each $\Delta \in \mathbf{Set}$, a symmetric monoidal closed category $C\Delta$ is given, and any function $f : \Delta \to \Delta'$ induces a strict symmetric monoidal closed functor $Cf : C\Delta' \to C\Delta$, performing renaming of indexes.

Upon this indexed symmetric monoidal closed categories, we introduce a categorical structure that models the $!_f$ modality. We call it *indexed linear exponential comonad*. This is a generalization of the *semiring-graded linear exponential comonad* studied in [13,22]. Our generalization replaces the semiring with $\mathbf{Idx}$, which may be regarded as a many-object pseudo-semiring (Proposition 2.1).

We write $[\mathbb{C}, \mathbb{D}]_l$ for the category of symmetric lax monoidal functors from $\mathbb{C}$ to $\mathbb{D}$ and monoidal natural transformations between them. We equip it with the pointwise symmetric monoidal structure $(\dot{I}, \dot{\otimes})$ given by $\dot{I}X = I$ and $(F \dot{\otimes} G)X = FX \otimes GX$ for $X \in \mathbb{C}$.

**Definition 4.1.** *An* indexed linear exponential comonad *(ILEC for short) over a set-indexed SMCC $C$ consists of:*

– *A collection of symmetric colax monoidal functors*

$$(D, w^{\Delta,\Delta'}, c^{\Delta,\Delta'}) : \mathbf{Idx}(\Delta, \Delta') \to [C\Delta', C\Delta]_l \quad (\Delta, \Delta' \in \mathbf{Set}).$$

*The symmetric lax monoidal structure of $Df$ is denoted by $m_f : I \to DfI$ and $m_{f,A,B} : DfA \otimes DfB \to Df(A \otimes B)$.*

– *Monoidal natural transformations $\epsilon^{\Delta} : D(\mathrm{i}_{\Delta}) \to \mathrm{Id}_{D\Delta}$ and $\delta_{g,f} : D(g \bullet f) \to Df \circ Dg$ satisfying axioms in Figure 3.*

– *$Cr' \circ Df \circ Cr = D(Jr \bullet f \bullet Jr')$ holds for any morphism $f$ in $\mathbf{Idx}$ and $r, r'$ in $\mathbf{Set}$ of appropriate type.*

The last axiom has two purposes: the equality $Cr'(DfA) = D(f \bullet Jr')A$ is to allow reindexing functions to act from outside, and the other equality $Df(CrA) = D(Cr \bullet f)A$ is to make $D$ invariant under internal reindexing of formulas. These equalities are tied up with the formula equivalence in (2.1) and the definition of reindexing at $!_f A$ in Definition 2.1, respectively. We postpone a concrete example of ILEC to Section 4.2.

### 4.1 Semantics of GBLL

We interpret a well-formed formula $\Delta \vdash A$ as an object $[\![\Delta \vdash A]\!] \in C\Delta$. This is done by induction on the structure of the formula. We assume that each atomic formula $a \in \mathcal{A}(\Delta)$ comes with its interpretation as an object $[a] \in C\Delta$.

$$[\![\Delta \vdash a \star r]\!] \stackrel{\text{def}}{=} Cr[a] \qquad [\![\Delta \vdash !_f A]\!] \stackrel{\text{def}}{=} Df[\![\Delta' \vdash A]\!]$$

$$[\![\Delta \vdash A \otimes B]\!] \stackrel{\text{def}}{=} [\![\Delta \vdash A]\!] \otimes [\![\Delta \vdash B]\!] \quad [\![\Delta \vdash A \multimap B]\!] \stackrel{\text{def}}{=} [\![\Delta \vdash A]\!] \multimap [\![\Delta \vdash B]\!]$$

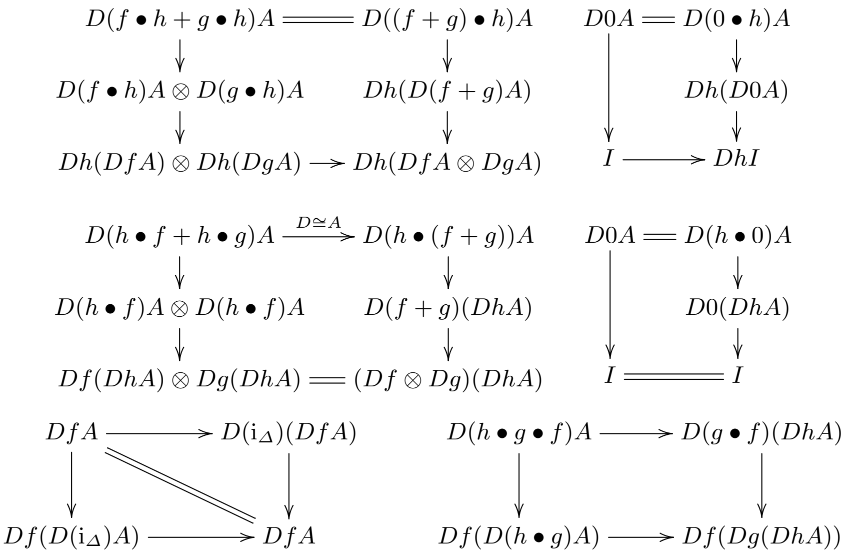

**Fig. 3.** Axioms of Indexed Linear Exponential Comonad

**Proposition 4.1.** *For any* $r \in \mathbf{Set}(\Delta, \Delta')$ *and well-formed formula* $\Delta' \vdash A$*, we have* $[\![\Delta \vdash A|_r]\!] = Cr[\![\Delta' \vdash A]\!]$.

**Proposition 4.2.** $[\![\Delta \vdash !_{Jr \bullet f} A]\!] = [\![\Delta' \vdash !_f (A|_r)]\!]$.

Each proof $\pi \triangleright \Delta \mid \Gamma \vdash A$ of GBLL is interpreted as a morphism $[\![\Delta \mid \Gamma \vdash A]\!] : [\![\Delta \vdash \Gamma]\!] \to [\![\Delta \vdash A]\!]$ in $C\Delta$. Here, for a sequence $\Gamma = C_1, \cdots, C_m$ of formulas, $[\![\Delta \vdash \Gamma]\!]$ denotes $[\![\Delta \vdash C_1]\!] \otimes \cdots \otimes [\![\Delta \vdash C_m]\!]$. We write out the interpretation only for the cases of modalities, because the other rules, Axiom, Exchange, Cut, $\otimes$(L, R) and $\multimap$(L, R) are interpreted similarly to the semantics of multiplicative intuitionistic linear logic. Fig. 4 shows the interpretation of rules related to $!_f$.

**Theorem 4.1.** *For a proof* $\pi \triangleright \Delta \mid \Gamma \vdash A$*, if* $\pi$ *has a reducible cut and reduces into* $\pi'$ *by a reduction step, then* $[\![\pi]\!] = [\![\pi']\!]$ *in* $C\Delta$.

### 4.2 Construction of an Indexed Linear Exponential Comonad

We present a construction of an indexed SMCCs $C : \mathbf{Set}^{op} \to \mathbf{SMCC}_{\mathrm{strict}}$ and an ILEC $D : \mathbf{Idx}(\Delta, \Delta') \to [C\Delta', C\Delta]_l$ over $C$ from a SMCC $\langle \mathbb{C}, \otimes, I, \multimap \rangle$, and a symmetric lax monoidal comonad $\langle V, m^V, m^V_{X,Y}, \epsilon, \delta \rangle$ on $\mathbb{C}$.

**Construction of Indexed SMCCs** First, for each index set $\Delta$, we define the category $\Delta \pitchfork \mathbb{C}$ to be the product of $\Delta$-many copies of $\mathbb{C}$. We represent objects and morphisms of this category by maps $\mathsf{X} : \Delta \to \mathrm{Obj}(\mathbb{C})$ and maps

$$\left[\!\!\left[\frac{\pi' \rhd \Delta \mid \Gamma \vdash B}{\Delta \mid \Gamma, !_{0_{\Delta,\Delta'}} A \vdash B}\right]\!\!\right] = [\![\Gamma, !_{0_{\Delta,\Delta'}} A]\!] \xrightarrow{\mathrm{id}\otimes w^{\Delta,\Delta'}_{[\![A]\!]}} [\![\Gamma]\!] \otimes I \xrightarrow{[\![\pi']\!]\circ\cong} [\![B]\!]$$

$$\left[\!\!\left[\frac{\pi' \rhd \Delta \mid \Gamma, A \vdash B}{\Delta \mid \Gamma, !_{\mathrm{id}} A \vdash B}\right]\!\!\right] = [\![\Gamma, !_{\mathrm{i}_\Delta} A]\!] \xrightarrow{\mathrm{id}\otimes \epsilon^{\Delta}_{[\![A]\!]}} [\![\Gamma]\!] \otimes [\![A]\!] \xrightarrow{[\![\pi']\!]} [\![B]\!]$$

$$\left[\!\!\left[\frac{\pi' \rhd \Delta \mid \Gamma, !_g A \vdash B \quad \sigma : f \Rightarrow g}{\Delta \mid \Gamma, !_f A \vdash B}\right]\!\!\right] = [\![\Gamma, !_f A]\!] \xrightarrow{\mathrm{id}\otimes (D\sigma)_{[\![A]\!]}} [\![\Gamma, !_g A]\!] \xrightarrow{[\![\pi']\!]} [\![B]\!]$$

$$\left[\!\!\left[\frac{\pi' \rhd \Delta \mid \Gamma, !_f A, !_g A \vdash B}{\Delta \mid \Gamma, !_{f+g} A \vdash B}\right]\!\!\right] = [\![\Gamma, !_{f+g} A]\!] \xrightarrow{\mathrm{id}\otimes c_{f,g}} [\![\Gamma]\!] \otimes ([\![!_f A]\!] \otimes [\![!_g A]\!]) \xrightarrow{[\![\pi']\!]\circ\cong} [\![B]\!]$$

$$\left[\!\!\left[\frac{\pi' \rhd \Delta, g \mid !_{g_1} A_1, \cdots, !_{g_k} A_k \vdash B}{\Delta \mid !_{g_1 \bullet f} A_1, \cdots, !_{g_k \bullet f} A_k \vdash !_f B}\right]\!\!\right] = \bigotimes_i [\![!_{g_i \bullet f} A_i]\!] \xrightarrow{\bigotimes_i \delta_{g_i, f, [\![A_i]\!]}} \bigotimes_i Df([\![!_{g_i} A_i]\!]) \xrightarrow{m_{f,\cdots [\![!_{g_i} A_i]\!] \cdots}} Df\left(\bigotimes_i [\![!_{g_i} A_i]\!]\right) \xrightarrow{Df([\![\pi']\!])} [\![!_f B]\!]$$

Here, 1) $[\![A]\!]$ denotes $[\![\Delta \vdash A]\!]$ for each well-formed formula $\Delta \vdash A$. 2) $\pi'$ denotes the proof of the premise of each rule.

**Fig. 4.** Interpretations of Modal Rules.

$\mathsf{f} : \Delta \to \mathrm{Mor}(\mathbb{C})$, respectively. Since SMCCs are closed under products, $\Delta \pitchfork \mathbb{C}$ is a SMCC by the component-wise tensor product and internal hom:

$$\mathsf{I}(d) \overset{\mathrm{def}}{=} I, \quad \mathsf{X} \mathbin{\dot{\otimes}} \mathsf{Y}(d) \overset{\mathrm{def}}{=} \mathsf{X}(d) \otimes \mathsf{Y}(d), \quad \mathsf{X} \mathbin{\dot{\multimap}} \mathsf{Y}(d) \overset{\mathrm{def}}{=} \mathsf{X}(d) \multimap \mathsf{Y}(d)$$

We then define the indexed SMCCs $C$ by $C\Delta \overset{\mathrm{def}}{=} \Delta \pitchfork \mathbb{C}$.

**Folding Product** We next introduce the *folding product* functor $\mathsf{T}$; we later compose it with the symmetric lax monoidal comonad $V$ so that we can derive various ILECs over $C$. Note that $\mathsf{T}$ itself *is* also an ILEC; set $V = \mathrm{Id}$. The type of $\mathsf{T}$ is $\Delta^* \times (\Delta \pitchfork \mathbb{C}) \longrightarrow \mathbb{C}$, and is defined by

$$\mathsf{T}(i_1 i_2 \cdots i_n, \mathsf{A}) \overset{\mathrm{def}}{=} \mathsf{A}(i_1) \otimes \mathsf{A}(i_2) \otimes \cdots \otimes \mathsf{A}(i_n), \quad \mathsf{T}((), \mathsf{A}) \overset{\mathrm{def}}{=} I$$

On morphisms, $\mathsf{T}$ maps a list permutation in the first argument to the symmetry morphism in $\mathbb{C}$. $\mathsf{T}$ is symmetric strong monoidal in each argument. Moreover, each strong monoidal structure interacts well with each other, concluding that it becomes a multi-symmetric strong monoidal functor in the sense of [21].

**Proposition 4.3.** *For $f \in \mathbf{Idx}(\Delta, \Delta')$ and $l = i_1 \cdots i_k \in \Delta^*$, let $f(l)$ denote $f(i_1) \cdots f(i_k)$. Then it holds $\mathsf{T}(f(l), \mathsf{A}) \simeq \mathsf{T}(l, \mathsf{T}(f(\_), \mathsf{A}))$ and this isomorphism is natural for $\mathsf{A}$.*

*Remark 4.1.* Usually the !-modal formula $!A$ in linear logic is interpreted by the object consisting of many copies of the same data (referred as *uniformity* of $!A$ [8]). We leave the development of uniform folding product as a future work.

**Construction of ILEC** We now compose the folding product functor with the symmetric lax monoidal comonad $V$, to derive another ILEC. Let $\Delta, \Delta'$ be index sets. We define a symmetric strong (hence colax) monoidal functor $D : \mathbf{Idx}(\Delta, \Delta') \longrightarrow [C\Delta', C\Delta]_l$ by

$$Df\mathsf{A}(i) \stackrel{\text{def}}{=} \mathsf{T}(f(i), V \circ \mathsf{A}) \quad Df\mathsf{p}(i) \stackrel{\text{def}}{=} \mathsf{T}(f(i), V\mathsf{p}) \quad D\alpha\mathsf{A} \stackrel{\text{def}}{=} \mathsf{T}(\alpha, V \circ \mathsf{A}). \quad (4.1)$$

Here, $\mathsf{A} \in \Delta' \pitchfork \mathbb{C}$, and $\mathsf{p}$ and $\alpha$ are morphisms in $\Delta' \pitchfork \mathbb{C}$ and $\mathbf{Idx}(\Delta, \Delta')$, respectively. We also define a helper morphism $\gamma^l_{\mathsf{A}} : \mathsf{T}(l, V \circ \mathsf{A}) \to V\mathsf{T}(l, \mathsf{A})$ for $(l_1 \cdots l_k) \in \Delta^*$ and $\mathsf{A} \in \Delta \pitchfork \mathbb{C}$. It is the multiple composite of $m_{A,B}$:

$$V\mathsf{A}(l_1) \otimes \cdots \otimes V\mathsf{A}(l_k) \to V\left(\mathsf{A}(l_1) \otimes \cdots \otimes \mathsf{A}(l_k)\right).$$

It is routine to verify that this morphism is monoidal natural on $l$ and $\mathsf{A}$.

Two monoidal natural transformations $\epsilon : D\mathrm{i}_\Delta \to \mathrm{Id}_{\Delta \pitchfork \mathbb{C}}$ and $\delta_{g,f} : D(g \bullet f) \to Df \circ Dg$ are defined by:

$$\epsilon_{\mathsf{A},i} : \mathsf{T}(i, V \circ \mathsf{A}) = V\mathsf{A}(i) \quad (4.2)$$

$$\begin{aligned} \delta_{g,f;\mathsf{A};i} : &\mathsf{T}((g \bullet f)(i), V \circ \mathsf{A}) \xrightarrow{\sim} \mathsf{T}(f, \mathsf{T}(g(\_), V \circ \mathsf{A})) \\ &\xrightarrow{\mathsf{T}(f, \mathsf{T}(g(\_), \delta_{\mathsf{A}}))} \mathsf{T}(f, \mathsf{T}(g(\_), V \circ V \circ \mathsf{A})) \xrightarrow{\mathsf{T}(f, \gamma^{g(\_)}_{\mathsf{A}})} Df(Dg\mathsf{A})(i). \end{aligned} \quad (4.3)$$

**Theorem 4.2.** *The symmetric colax monoidal functor $D$ (4.1) and monoidal natural transformations $\epsilon, \delta$ (4.2,4.3) determine an ILEC over $C$.*

### 4.3 GBLL Semantics by Realizability Category

Hofmann et al., and also Dal Lago et al. employ a *realizability semantics* to show that the complexity of BLL proof reductions belongs to P-time [19,10]. In this section we compare their semantics and the simple semantics of GBLL constructed in the previous section.

We instantiate $\mathbb{C}$ in the previous section with the realizability category over a BCI algebra $(A, \cdot)$, which is a combinatory algebra based on $B, C, I$-combinators; see e.g. [2,20]. We then form the realizability category $\mathbf{Ass}(A)$ by the following data: an object is a function $f$ into $P^+A$, where $P^+$ is the nonempty powerset construction, and a morphism from $f$ to $g$ is a function $h : \mathrm{dom}\, f \to \mathrm{dom}\, g$ with the following property: there exists an element $e \in A$ such that for any $x \in \mathrm{dom}\, f$ and $a \in f(x)$, we have $e \cdot a \in g(h(x))$. The category $\mathbf{Ass}(A)$ is symmetric monoidal closed; see e.g. [20, Proposition 4]. The tensor product of $f$ and $g$ is given by $(f \otimes g)(x, y) = \{u \boxtimes v \mid u \in f(x), v \in g(y)\}$, where $u \boxtimes v$ is the BCI-algebra element corresponding to $\lambda x.xuv$ [20, Section 2].

Next, let $\Delta$ be a set and consider the power category $\Delta \pitchfork \mathbf{Ass}(A)$. Under the axiom of choice, $\Delta \pitchfork \mathbf{Ass}(A)$ is equivalently described as follows: an object is a family of functions $\{f_i\}_{i\in\Delta}$ into $P^+A$, and a morphism from $\{f_i\}_{i\in\Delta}$ to $\{g_i\}_{i\in\Delta}$ is a family of functions $\{h_i : \mathrm{dom}\, f_i \to \mathrm{dom}\, g_i\}_{i\in\Delta}$ with the following property: there exists a function $e : \Delta \to A$ such that for any $i \in \Delta$, $x \in \mathrm{dom}\, f_i$ and $a \in f_i(x)$, we have $e(i) \cdot a \in g_i(h_i(x))$.

This power category is quite close to the realizability category introduced in [19, Section 4] and [10, Section 4]. A membership statement $a \in f_i(x)$ for an object $\{f_i\}_{i\in\Delta} \in \Delta \pitchfork \mathbf{Ass}(A)$ corresponds to a realizability statement $i, a \Vdash x$ in the realizability category (see [19]). The major difference between these categories is twofold: 1) In the realizability category, a computability constraint is imposed on $e : \Delta \to A$ to achieve the characterization of P-time complexity. 2) Objects in the realizability category are limited to $\Delta \pitchfork \mathbf{Ass}(A)$-objects such that all $f_i$ share the common domain. This is to synchronize with the set-theoretic semantics *ignoring* resource polynomials [19, Section 3] [10, Section 3].

We compute the bounded !-modality using the folding product ILEC $\mathsf{T}$ with respect to the indexed SMCC $(\_) \pitchfork \mathbf{Ass}(A)$. Let $F$ be a finite set of variables, $x \notin F$ be a resource variable, $p$ be a resource polynomial and $\mathscr{C}$ be a constraint set under $F$. For any object $\mathsf{X}$ in $V_{\mathscr{C}\cup\{v\le p\}}(F\cup\{v\}) \pitchfork \mathbf{Ass}(A)$, the folding product $\mathsf{T}([v \le p]_{(F,\mathscr{C})}, \mathsf{X})$ is an object in $V_{\mathscr{C}}(F) \pitchfork \mathbf{Ass}(A)$ satisfying

$$\begin{aligned}&\mathsf{T}([v < p]_{(F,\mathscr{C})}, \mathsf{X})(i)\\&= \lambda(x_0, \cdots, x_{[\![p]\!]i-1})\ .\ \{a_0 \otimes \cdots \otimes a_{[\![p]\!]i-1} \mid a_j \in \mathsf{X}(i\{v \mapsto j\})(x_j)\}\end{aligned} \tag{4.4}$$

This is different from the modality over the realizability category introduced in [19, Definition 16] and [10, Definition 4.6]:

$$(!_{v<p}\mathsf{X})(i) = \lambda x\ .\ \{a_0 \otimes \cdots \otimes a_{[\![p]\!]i-1} \mid a_j \in \mathsf{X}(i\{v \mapsto j\})(x)\};$$

it only takes a single argument. This is again because their realizability semantics is designed to synchronize with the set-theoretic semantics ignoring resource polynomials — especially it interprets $[\![!_{x\le p}A]\!] = [\![A]\!]$. On the other hand, the bounded quantification computed in (4.4) does *not* ignore resource polynomials and indexing, as the domain of (4.4) is the index-dependent product $\prod_j \mathrm{dom}(\mathsf{X}(i\{v \mapsto j\}))$. From this, we conjecture that the semantics of $\mathsf{BLL}$ using the ILEC $\mathsf{T}$ over $(\_) \pitchfork \mathbf{Ass}(A)$ realizes an *index-dependent* set-theoretic semantics of $\mathsf{BLL}$ — we leave this semantics as a future work.

## 5 Conclusion and Related Work

We introduced $\mathsf{GBLL}$, a generalization of Girard et al.'s $\mathsf{BLL}$. We analyzed the complexity of cut-elimination in $\mathsf{GBLL}$, and gave a translation from $\mathsf{CBLL}$, an extension of $\mathsf{BLL}$ with constraints to $\mathsf{GBAL}^+$. We then introduced ILEC as a categorical structure for interpreting the !-modality of $\mathsf{GBLL}$. The ILEC is a **Idx**-graded linear exponential comonad interacting well with a specified indexed SMCCs. We gave an elementary construction of ILEC using the folding product, and a technique to derive its variants by inserting symmetric monoidal comonads. We gave the semantics of BLL using the folding product on the category of assemblies of a BCI-algebra, and related with the realizability category studied in [19,10].

Girard's BLL has a great influence on the subsequent development of indexed modalities and implicit complexity theory [16]. Hofmann and Scott introduced the realizability technique to BLL and semantically proved that BLL characterizes P-time complexity [19]. Their work was further enriched and studied by Dal Lago and Hofmann [10]. Gaboardi combined the !-modality involving variable binding with PCF and showed that the combined system is relatively complete [24].

Bucciarelli and Ehrhard's *indexed linear logic with exponential* [9] is one of the closest systems to GBLL. However, the type of the !-modality is different: their system derives $\Delta \vdash !_f A$ from $\Delta' \vdash A$ and an *almost injective function* $f : \Delta' \to \Delta$; it is a function where each $f^{-1}(i)$ is finite. To relate their system and GBLL, let us use the finite powerset construction $P_{\mathrm{fin}}$ and convert $f$ into its inverse $f^{-1} : \Delta \to P_{\mathrm{fin}}(\Delta')$. This exhibits the similarity with GBLL: GBLL relaxes $P_{\mathrm{fin}}$ to $(\_)^*$, and takes the inverse as the parameter for the !-modality. The novelty of this work to [9] is that a categorical axiomatization for the $!_f$ modality is identified as an extension of the graded linear exponential comonads [7,22]. Another novelty is to show that GBLL is enough to encode BLL.

As described in Section 1, the simple form of !-modality $!_r A$ is also widely used in various type systems and programming languages. Examples include: INTML [30], coeffect calculus [28,7] and its combination with effect systems [13], Granule language [26], bounded linear type system [14,26], type systems for the analysis of higher-order model-checking [18,17], a generic BLL-like logic $\mathrm{B}_{\mathcal{S}}\mathrm{LL}$ over semirings [6], Fuzz type system for function sensitivity and differential privacy [29,12,3], and many more. A combination of $!_r A$ with dependent type theory called QTT is also introduced in [25] and [4]. Among these systems, each of [12,26,1] supports 1) full universal and existential, 2) full universal and 3) partial universal quantification over grades, respectively.

The categorical structure corresponding to the simple form of !-modality appears in [7,13,22] and is identified as *semiring-graded linear exponential comonad*. Breuvart constructed various examples of semiring-graded linear exponential comonads on relational models of linear logic [6] using his *slicing* technique. In this work we replaced semirings to **Idx**, which may be seen as a multi-object pseudo-semiring. In the study of graded monad, Orchard et al. generalize the grading structure from ordered monoids to 2-categories [27]. The main difference from this work is that their generalized graded monad is defined over a single category, while an ILEC is defined over an *indexed* SMCCs.
